# Effects of linear and change of direction high-intensity interval training on physical performance of elite female soccer players

**DOI:** 10.5114/biolsport.2024.134761

**Published:** 2024-03-06

**Authors:** Mima Stanković, Nebojša Trajković, Draženka Mačak, Dušan Đorđević, Anja Lazić, Zoran Milanović

**Affiliations:** 1 Faculty of Sport and Physical Education, University of Niš, Niš, Serbia; 2 Faculty of Sport and Physical Education, University of Novi Sad, Novi Sad, Serbia; 3 Science and Research Centre of Koper, Koper, Slovenia; 4 Faculty of Sports Studies, Incubator of Kinanthropological Research, Masaryk University, Brno, Czech Republic

**Keywords:** HIIT, Endurance, Interval, Women, Running, Power, Football

## Abstract

The aim of this study was to determine the effects of linear and change of direction high-intensity interval training (HIIT) on physical performance in elite female soccer players. Thirty elite female soccer players (age: 19.6 ± 4.6 years; height: 166.5 ± 4.8 cm; body weight: 60.5 ± 8.3 kg; BMI: 21.9 ± 2.9 kg/m^2^) were randomly allocated to HIIT linear (HIIT_LIN_) and HIIT change of direction (HIIT_COD_) training groups. The HIIT_LIN_ group performed linear running for 15, 20 or 25 s by keeping constant pace during the entire distance. In contrast, the HIIT_COD_ group performed three changes of direction (COD) with a 180° turn during each interval run at 15, 20 or 25 s. Physical performance was assessed using sprinting, agility, vertical jumps, repeated sprint ability (RSA) and 30–15 Intermittent Fitness Test (30–15 IFT). In both groups, all physical performance measurements improved (p ≤ 0.05), except RSAbest in HIIT_LIN_ (p = 0.45). Both interventions significantly improved speed over 10 m, 20 m, 30 m, Pro-agility, Zig-zag, RSAavg, fatigue index, maximal oxygen uptake, and velocity at 30–15 IFT, while moderate improvements were observed in countermovement jump (CMJ), CMJ with arm swing and squat jump. However, HIIT_COD_ did not achieve superior improvements in any of the aforementioned measurements compared to HIIT_LIN_. Based on the obtained results, we concluded that different types of HIIT training have a positive effect on physical performance in elite female soccer players.

## INTRODUCTION

Elite female soccer players cover around 9–11 km [1] of total distance during the game and spend 1.2–2.7 km performing high-intensity activities [2]. Furthermore, female soccer is characterized by frequent high-intensity activities throughout the game, which requires a high level of players’ functional and motor skills. More precisely, speed, strength, agility, and endurance are crucial to perform a variety of elements such as jumping, accelerations, decelerations and changes of direction (COD) activities [3]. In order to repeat these actions and keep overall performance at the highest level, female soccer players must develop a very high level of aerobic and anaerobic capacity. However, little is known about the most effective strategy to simultaneously improve both aforementioned parameters in female soccer players because studies about female soccer players accounted for only 20% of all soccer research, while only 15% of them were dedicated to training and performance in elite female soccer players [4].

To date, some studies have confirmed that high-intensity interval training (HIIT) is one of the most effective methods for developing and improving aerobic and anaerobic capacity in team sports athletes [5]. In addition, HIIT training is potentially recognized as the most time-efficient method to achieve adaptive exercise goals [6]. Therefore, HIIT is considered to be the optimal type of training to improve both aerobic and anaerobic capacity and appears to be an effective method of fitness training for female soccer players [7]. However, the effects of HIIT on physical performance in female soccer players have received little research attention with only a few studies evaluating the effects of heart rate vs. speed-based HIIT, mixed methods HIIT and short-term HIIT in amateur [8], college [9) and youth [10] female soccer players, respectively. Specifically, in a recent study [8] both heart rate-based and speed-based HIIT induced meaningful improvements in aerobic and anaerobic capacity in amateur female soccer players. In addition, Rowan et al. [9] observed that short-term HIIT had positive effects on V̇O_2max_ (3.4–4.7%) in female college soccer players, while Wright et al. [10] reported very likely moderate and trivial improvements of Yo-Yo IR and the 20 m sprint test, respectively (effect size (ES) = 0.38–1.39), after six weeks in regional-level soccer players. Moreover, Wright et al. [10] also observed slightly improved agility (2.4%) and repeated sprint (1.7–6.5%) performance in young female soccer players (age 13.4 ± 1.5 years). However, the effects of HIIT on physical performance in elite female soccer players remain unproven due to the scarcity of research attention.

All the aforementioned HIIT interventions were based on linear running despite practitioners’ tendency to apply a more specific type of HIIT training which is similar to the specific game conditions [11]. More precisely, practitioners prefer HIIT training with a change of direction (HIIT_COD_), which is more specific, in terms of movement patterns, to the female soccer match. In this vein, an approach to overcome this limitation in applied HIIT methods may be drawn from the combination of COD speed and HIIT research. More precisely, Ashton & Twist [12] found that the combination of HIIT and COD training leads to improved aerobic and anaerobic capacity in male soccer players. In addition, Teixeira et al. [13] concluded that HIIT with three directional changes per running bout achieved superior improvements in aerobic capacity, running economy, and repeated sprint ability (RSA) compared to one directional change in female futsal players. However, the effects of HIIT_COD_ on physical performance in female soccer players are still unknown. In addition, there is no study comparing the effects of linear based HIIT (HIIT_LIN_) and HIIT_COD_ on physical performance in female soccer players. Therefore, the aim of this study was to determine the effects of linear and change of direction HIIT training (HIIT_COD_ vs. HIIT_LIN_) on physical performance in elite female soccer players. We hypothesized that both HIIT_LIN_ and HIIT_COD_ produce similar improvements in linear running performance while HIIT_COD_ will be superior in COD movements.

## MATERIALS AND METHODS

### Participants

Thirty elite female soccer players (age: 19.6 ± 4.6 years; height: 166.5 ± 4.8 cm; body weight: 60.5 ± 8.3 kg; BMI 21.9 ± 2.9 kg/m^2^) participated in this study. The participants were randomly allocated to HIIT_LIN_ (N = 15; age: 19.2 ± 4.2 years; height: 165.5 ± 5.6 cm; body weight: 60.3 ± 8.2 kg; training experience: 6.3 ± 4.4 years) and HIIT_COD_ (N = 15; age: 20.0 ± 5.0 years; height: 167.3 ± 4.3 cm; body weight: 63.6 ± 9.1 kg; training experience: 6.1 ± 4.7 years) after baseline measurements according to the procedure described by Suresh [14]. The inclusion criteria were as follows: individuals over the age of 15, who are competing at the highest level of soccer competition and who have trained with at least five training sessions per week. The exclusion criteria were: anyone with cardiovascular, respiratory, or other diseases, those undergoing rehabilitation, individuals who have had some kind of distortion in the last three months, as well as participants who had anterior cruciate ligament surgery in the previous year. All participants and their guardians were informed about the experimental design and testing procedures before providing written information and consent to participate. All study procedures were approved by the Human Research Ethics Committee at the University of Nis and followed the Helsinki Declaration guidelines (ref no. 04–1085/2). The club management and the participants were acquainted in detail with the experimental research.

### Procedures

Measurements of the physical performance of all participants at the initial and the final measurements were conducted in a multidisciplinary diagnostic centre, as well as in the field with natural grass and lasted for two days. Measurements during both days were performed in the period 9–11 a.m. All tests were performed in the same order during initial and final measurements. The data were automatically stored in the computer by the direct method of input from the instrument measured in most cases, and only when there was no software solution would the results be manually entered on pre-prepared lists for meters.

All participants performed a standard 20 min warm-up protocol consisting of jogging (6 min), stretching (5 min), progressive running (3 min), COD (3 min) and low-intensity plyometric jumps (3 min). Afterwards, participants performed vertical jumps (CMJ, CMJ with arm swing (CMJA), SJ) and RSA tests. On the second day after the same warm-up routine, participants performed additional physical performance tests as follows: 30 m sprint with time recorded at 10 m and 20 m, Pro-agility test, Zig-zag test, 9-6-3-6-3 sprint, 30–15 Intermittent Fitness Test (30–15 IFT). The same order of testing and procedure was performed by all participants at the initial and the final measurement.

### Speed (running 0–30 m)

This study used 10 m, 20 m, and 30 m linear sprinting tests. The running speed was determined using three pairs of photocell infrared timing gates (Microgate, Polifemo Radio Light, Bolzano, Italy). Before testing, the players were familiarized with the procedures. Thirty cm behind the first pair of photocells was marked as the starting point. Participants were instructed to run as quickly as possible over the 30 m distance from a standing start. The timer was automatically activated as participants crossed the first gate at the starting line with split times at 10 m and 20 m. Acceleration was evaluated using the time to cover the first 10 m of the 30 m test. The participants performed three trials with at least 3 min rest between them [15]. The best performance of the three trials was used for further analysis.

### Pro-agility test

The ability to change direction laterally to the right and left was measured by the Pro-agility test (5-10-5 m test). The running speed (5-10-5 m) was determined using photocell timing gates (Microgate, Polifemo Radio Light, Bolzano, Italy). The participants’ starting position was in the middle of the 10 m separated cones and the timer was automatically activated as the participants crossed the first gate at the starting line. Each participant had to choose the side for the sprint (right or left) on the first attempt, while the opposite direction was used in the second attempt. The main task was to run laterally (left/right) to the nearest cone (5 m), then run (left/right) to the next cone (10 m), and another one (left/right) which was the starting point (5 m). The time was automatically stopped when the participant re-crossed the starting line. The participants performed three trials with at least 3 min rest between them. The best performance of the three trials was used for further analysis. The test validity and reliability have been confirmed elsewhere [16].

### Zig-zag test

The Zig-zag test measured running agility based on direction changes. The course consisted of four 5 m sections (20 m in total) with cones at a 100° and two pairs of photocells (Witty, Microgate, Bolzano, Italy) and required participants to slow down and accelerate around each cone as quickly as possible, from separated starting and finishing points. Before testing, the players were familiarized with the procedures. Starting from a standing position with the front foot 30 cm behind the first pair of timing gates, the players were instructed to complete the test as quickly as possible until they crossed the second pair of timing gates. The participants had a maximum of three attempts with an interval of 3 min of rest in between. The test validity and reliability have been confirmed elsewhere [17].

### 9-6-3-6-9 sprint (with 180° turns)

The players started after the signal and ran 9 m. Touching the line with one foot, they made a turn of 180° to the left or the right. The players then ran 3 m to the next line, made another 180° turn and ran 6 m forward. Then they made another 180° turn and ran 3 m forward, before making the last turn and the final 9 m run to the finish line. In addition, the main participants’ aim was to perform the previously described test as fast as possible. The distance the players covered during this test was 21 m. The participants had a maximum of three attempts with an interval of 3 min of rest in between. The validity and reliability of the test have been confirmed elsewhere [18].

### Vertical jumps (CMJ, CMJA, SJ)

Three types of vertical jumps were used to evaluate jump height (cm) – countermovement jump (CMJ), CMJ with arm swing (CMJA) and squat jump (SJ). Two photoelectric cells (Optojump, Microgate, Bolzano, Italy) were used to measure flight time between take-off and landing (cm). Before performing the tests, the participants were instructed to jump as high as they could. While performing the CMJ test, the participants had to put their hands on the hips and, on the previously set signal, flex the knees (approximately 90°) in order to jump upward without moving their hands from the hips. The CMJA test was performed the same way as the CMJ test, except the hands were free during all the phases of the maximum jump. The SJ test was performed with knees flexed at 90° and hands on the waist, as the maintained starting position (3 s), in order to jump upward without moving the hands from the hips. Additionally, for the jump to be correctly conducted, the participants had to avoid any knee or trunk countermovement. Each jump was completed after three trials with 1 min passive rest and 3 min passive rest between every test. For each test, the best result (cm) was taken into consideration. The validity and reliability of all previously described tests have been confirmed elsewhere [19].

### Repeated sprint ability (RSA)

The repeated sprint ability (RSA) test consisted of six 40 m sprints separated by 20 s of passive recovery. Two pairs of infrared photocell timing gates (Microgate, Polifemo Radio Light, Bolzano, Italy) were set up at the start, and two in a straight line at a distance of 20 m from the starting line. The participants began the test 30 cm behind the starting line. When a participant was ready, they ran through the timing gates to a line marked on the track (20 m from the starting line). When the participants’ foot reached the line at the 20 m mark, they turned as quickly as they could and ran back to the starting line. Then, the time was paused when the participant re-crossed the starting line. During testing, athletes were verbally instructed to take the starting position 30 cm behind the starting line 5 s before each sprint and a three second countdown was provided to start again. The procedure was repeated 6 times, with 20 s of active recovery between the runs. The results were added together and two results were recorded – the average time (RSAavg) and the total time (RSAtime) for the run section expressed in seconds with an accuracy of 1/100. Given that the RSA test caused fatigue in participants after a certain number of repetitions, the fatigue index in them was determined. The calculation of this parameter was done by dividing the difference between the best and the worst time with the best time and multiplying it by 100. The validity and reliability have been confirmed elsewhere [20]. In addition, the estimate of the fatigue index was calculated by the formula:

FI = 100 × [(best time (s) – worst time (s)) / best time (s)]

where FI is the fatigue index, s is time on the RSA test expressed in seconds

### 30–15 Intermittent Fitness Test

The 30–15 IFT test was performed by placing cones 40 m apart from the beginning to the end and creating two three-metre zones in the middle of the testing area which helped guide the athletes to adjust and maintain their speed. Each player had their own line to run back and forth between 2 lines at a pace dictated by an auditory signal. The audio signal (beep) determined the running speed which started at 8 km/h for 30 s, with an increase of 0.5 km/h at each level (at 45 s). During the recovery period, the participants walked towards the nearest line, depending on where the previous run ended. During the 30–15 IFT, participants were verbally encouraged and instructed to cover as much distance as possible. The test ended when a participant was no longer able to maintain the imposed running speed or when she was unable to reach the 3 m zone around each line at the moment of the audio signal for 3 executive times. From the 30–15 IFT results, the indirect estimate of the maximum oxygen consumption in relative and absolute values was obtained [21]. The validity and reliability of this test have been confirmed elsewhere [21].

### Training protocol

The training programme was performed during the pre-season period with two HIIT sessions per week (Tuesday and Thursday) and lasted six weeks. Both experimental groups were supervised by at least one of the investigators during the intervention period. In addition, the participants also had five regular training sessions within the club consisting of technical and tactical drills. HIIT training sessions were separated by 48 h to allow sufficient recovery. All HIIT sessions during interventions were performed outdoors on the field with natural grass. Participants performed standardized warm-ups consisting of moderate-intensity jogging (5 min), static and dynamic stretching (5 min), passing and receiving drills (5 min) and acceleration running (2 min) prior to the HIIT session. Interval distances for each participant were individually adjusted according to each participant’s final speed reached at the end of the 30–15 Intermittent Fitness Test (VIFT) [6], which was performed at baseline and repeated every third week in order to update the training speed.

Each session during the entire training programme involved work intervals of running effort at an intensity of 100% VIFT interspersed by an equivalent amount of passive recovery (0% VIFT). Training load was gradually increased from week 1 to week 6. While training intensity was performed at 100% VIFT, total training workload was calculated as arbitrary training units (ATU) [22]. The summary of the training plan and load for HIIT_LIN_ and HIIT_COD_ is presented in Table 1. Briefly, the HIIT_LIN_ group performed linear running at 15, 20 or 25 s by keeping a constant pace during the entire distance. In contrast, the HIIT_COD_ group performed three COD of 180° turn during each interval running at 15, 20 or 25 s. The individual interval distance for the HIIT_COD_ group was reduced by 7% due to three turns of 180° which required additional energy. The aforementioned correction is based on the Laursen & Buchheit [6] recommendation that excess energy consumption should be compensated by reducing the distance by 2–3% for each COD compared to a straight line.

**TABLE 1:** Detailed presentation of the experimental programme for both groups (LIN and COD) lasting 6 weeks

Week	Interval (s)	Intensity (%) (work/rest)	Repetition	Series	Total duration (min)	Training load (ATU)
1–2	15 × 15	100/0	8	3	≈18	18.000
3–4	20 × 20	100/0	8	3	≈22	24.000
5–6	25 × 25	100/0	8	3	≈26	30.000

**Legend:** HIIT – high-intensity interval training, VIFT – final speed reached at 30–15 intermittent fitness test, TL – training load, LIN – linear, COD – change of direction

Participants started interval running from a standing position and followed audio signals to commence each subsequent run for the entire session. Upon completing each interval, participants immediately decelerated and were instructed to walk slowly to the next starting point and passively stand to await commencement of the next running interval. Every participant completed the running intervals in their own lane with fixed distances according to the calculated individualized VIFT. In addition to arbitrary training units, the rate of perceived exertion (RPE) scale was used to monitor training load of each HIIT session and provide greater insight into the training stresses [7].

ATU = [work intensity + rest intensity) / 2] × number of repetitions × number of series × duration

Example of HIIT training load calculation for 1–2 weeks: [(100 + 0) / 2] × 8 × 3 × 15 = 18 000 ATU

### Statistical analysis

G*power 3.1 power analysis software (Heinrich-Heine-University, Düsseldorf, Germany) determined the minimum total sample size (N-30) given the critical F = 4.60, effect size f = 0, P = 0.05, groups and time points = 2, and correlation among the measurements = 0.50. Data are expressed as mean [95% confidence interval] unless otherwise stated. The assumptions of normality and homogeneity of variances and covariances were verified using the Kolmogorov–Smirnov and Leven’s and Box’s tests, respectively.

A 2 × 2 mixed ANCOVA (group × time) model estimated the 6-week effects of HIIT_LIN_ and HIIT_COD_ training programmes on study outcomes after controlling for mean-centred age (mean = 19.60 years) and BMI (BMI = 21.88 kg/m^2^) effects. We analysed the baseline differences between the groups in study outcomes using simple main effects of a group. A simple main effect of time evaluated the mean changes [95% CI] in each outcome from initial to final testing within the groups with Bonferroni adjusted p values and 95% CIs. A time by group interaction effect tested whether mean changes [95% CI] in each outcome after 6 weeks depended on whether players performed HIIT with or without change of direction. Mean changes [95% CI] are based on estimated marginal means that are adjusted for mean-centred covariates appearing in the model. Cohen’s d effect sizes (ES) were also calculated to determine the magnitude of the group differences in physical fitness. ES magnitude was interpreted as: trivial = < 0.20; small = 0.2–0.59; moderate = 0.60–1.19; large = 1.20–1.99; and very large = > 2.0. We used SPSS Statistics version 23.0 (IBM SPSS Statistics for Windows, Armonk, NY: IBM) and GraphPad Prism version 8.0 (GraphPad Software, San Diego, California USA) to analyse and plot the data obtained, respectively.

## RESULTS

**FIG. 1:**
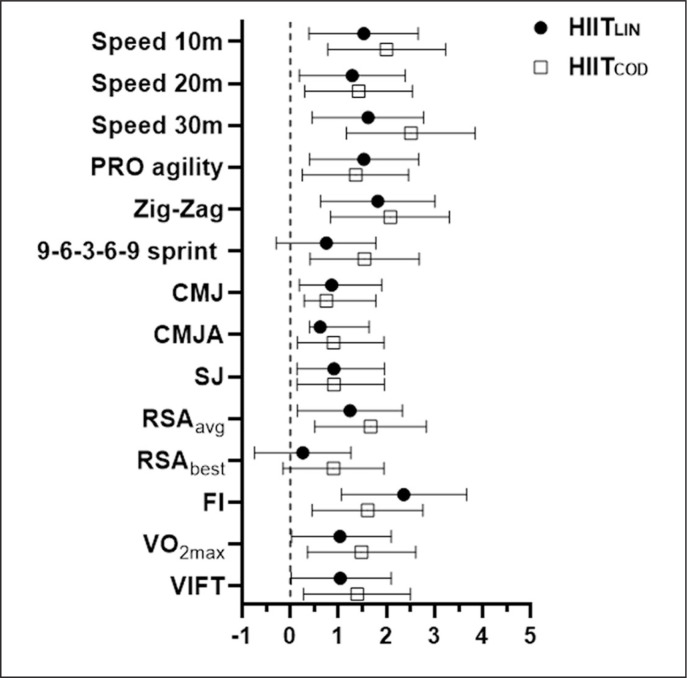
Effect size (ES) for all included variables of physical performance.

Baseline mean values of all study outcomes were similar across the groups (p ≤ 0.05). There was no time × group interaction (p ≤ 0.05) following 6 weeks of training (Table 2). In both groups mean performance of all tests significantly improved after 6 weeks (p ≤ 0.05), except RSAbest in HIIT_LIN_ (p = 0.4). We observed large improvements of HIIT_LIN_ and HIIT_COD_ mean performance of speed over 10 m (HIIT_LIN_ vs. HIIT_COD_: ES = 1.24 vs. 1.61), 20 m (HIIT_LIN_ vs. HIIT_COD_: ES = 1.21 vs. 0.95), and 30 m (HIIT_LIN_ vs. HIIT_COD_: ES = 1.55 vs. 1.28), Pro-agility (HIIT_LIN_ vs. HIIT_COD_: ES = 1.56 vs. 1.46), Zig-zag (HIIT_LIN_ vs. HIIT_COD_: ES = 1.47 vs. 1.59), RSAavg (HIIT_LIN_ vs. HIIT_COD_: ES = 1.13 vs. 0.92), FI (HIIT_LIN_ vs. HIIT_COD_: ES = 2.35 vs. 1.90), V̇O_2max_ (HIIT_LIN_ vs. HIIT_COD_: ES = -0.57 vs. -1.24), and VIFT (HIIT_LIN_ vs. HIIT_COD_: ES = -0.47 vs. -1.39), and medium improvements of HIIT_LIN_ and HIIT_COD_ mean performance CMJ, CMJA, and SJ (Figure 1). HIIT_COD_ increased performance of Speed 30 m to a significantly larger extent than HIIT_LIN_ (p ≤ 0.05).

**TABLE 2:** Physical performance parameters for HIIT_LIN_ (N = 15) and HIIT_COD_ (N = 15) groups before and after 6-week training intervention

Outcome	Group	Initial	Final	Mean changes [95% CI]	Δ (%)	ES	ANCOVA (group × time)	
							F	p
**Speed 10 m (s)†**	HIIT_LIN_	2.06 ± 0.11	1.86 ± 0.20	-0.20 [-0.31, -0.08]^**^	-9.7%	1.24	0.42	0.84
	HIITCOD	2.07 ± 0.13	1.89 ± 0.09	-0.18 [-0.30, -0.07]^**^	-8.7%	1.61		
**Speed 20 m (s)†**	HIIT_LIN_	3.57 ± 0.13	3.38 ± 0.18	-0.19 [-0.33, -0.05]*	-5.3%	1.21	0.01	0.92
	HIITCOD	3.54 ± 0.18	3.35 ± 0.22	-0.20 [-0.34, -0.06]**	-5.4%	0.95		
**Speed 30 m (s)†**	HIIT_LIN_	5.05 ± 0.28	4.68 ± 0.19	-0.36 [-0.54, -0.19]***	-7.3%	1.55	0.02	0.94
	HIITCOD	5.01 ± 0.29	4.68 ± 0.22	-0.34 [-0.52, -0.17]***	-6.6%	1.28		
**Pro-agility (s)†**	HIIT_LIN_	5.49 ± 0.24	5.16 ± 0.18	-0.32 [-0.48, -0.15] ***	-6.0%	1.56	0.01	0.94
	HIITCOD	5.40 ± 0.26	5.09 ± 0.15	-0.33 [-0.49, -0.16]***	-5.7%	1.46		
**Zig-zag (s)†**	HIIT_LIN_	6.31 ± 0.25	5.89 ± 0.31	-0.42 [-0.58, 0.27]***	-6.7%	1.47	0.73	0.40
	HIITCOD	6.24 ± 0.31	5.73 ± 0.33	-0.52 [-0.68, 0.36]***	-8.2%	1.59		
**9-6-3-6-9 (s)†**	HIIT_LIN_	8.75 ± 0.45	8.26 ± 0.31	-0.47 [-0.71, -0.23]***	-5.6%	1.27	0.53	0.47
	HIITCOD	8.78 ± 0.45	8.44 ± 0.28	-0.35 [-0.59, -0.11]**	-3.9%	0.91		
**CMJ (cm)**	HIIT_LIN_	24.01 ± 3.60	26.2 ± 2.26	1.99 [0.94, 3.03]***	9.1%	-0.73	0.60	0.44
	HIITCOD	22.62 ± 3.22	25.0 ± 2.62	2.55 [1.50, 3.59]***	10.5%	-0.81		
**CMJA (cm)**	HIIT_LIN_	26.65 ± 4.08	29.28 ± 2.82	2.52 [1.35, 3.69]***	9.9%	-0.75	0.03	0.87
	HIITCOD	25.42 ± 4.00	27.96 ± 2.88	2.66 [1.48, 3.83] ***	10.0%	-0.73		
**SJ (cm)**	HIIT_LIN_	22.33 ± 3.13	24.11 ± 2.19	1.71 [0.37, 3.04]*	8.0%	-0.66	1.75	0.20
	HIITCOD	20.83 ± 3.26	23.68 ± 3.01	2.93 [1.60, 4.27]***	13.7%	-0.90		
**RSA(avg)**	HIIT_LIN_	8.84 ± 0.36	8.39 ± 0.43	1.71 [0.37, 3.04]*	-5.1%	1.13	0.05	0.83
	HIITCOD	8.78 ± 0.47	8.37 ± 0.42	2.93 [1.60, 4.27]***	-4.7%	0.92		
**RSA(best)**	HIIT_LIN_	8.26 ± 0.29	8.21 ± 0.34	-0.05 [-0.31, 0.22]	-0.6%	0.16	0.03	0.86
	HIITCOD	8.26 ± 0.50	8.19 ± 0.33	-0.08 [-0.34, 0.18]	-0.8%	0.16		
**FI(%)†**	HIIT_LIN_	12.18 ± 3.76	5.18 ± 1.88	-6.71 [-9.08, -4.33]***	-57.5%	2.35	0.003	0.96
	HIITCOD	10.83 ± 4.41	4.50 ± 1.68	-6.62 [-8.99, -4.24]***	-58.4%	1.90		
V̇O**_2max_ (mL/kg/min)**	HIIT_LIN_	42.23 ± 2.82	43.86 ± 2.89	1.60 [0.08, 3.11]*	3.9%	-0.57	2.41	0.13
	HIITCOD	43.33 ± 1.60	46.52 ± 3.26	3.23 [1.71, 4.74]***	7.4%	-1.24		
**VIFT**	HIIT_LIN_	16.11 ± 1.19	16.75 ± 1.19	0.64 [0.17, 1.12] **	4.0%	-0.47	3.56	0.07
	HIITCOD	16.47 ± 0.54	17.75 ± 1.18	1.27 [0.79, 1.74] ***	7.8%	-1.39		

Δ (%) – percent change between initial and final measurement, ES – effect size,

† – reverse scoring; CMJ – counter movement jump; CMJA – counter movement jump free arms; SJ – squat jump; RSA – repeated sprint ability; FI – fatigue index; VIFT – final speed reached at 30–15 intermittent fitness test; statistical power;

* – significant difference between initial and final measurements (p ≤ 0.05);

** – significant difference between initial and final measurements p ≤ 0.01;

*** – significant difference between initial and final measurements p ≤ 0.01.

## DISCUSSION

The aim of this study was to determine the effects of HIIT training with and without change of direction on physical performance in elite female soccer players. Both interventions, HIIT_LIN_ and HIIT_COD_, significantly improved speed over 10 m, 20 m, 30 m, Pro-agility, Zig-zag, RSAavg, FI, V̇O_2max_, and VIFT, while moderate improvements were observed in CMJ, CMJA and SJ. However, HIIT_COD_ did not achieve superior improvements in any of the aforementioned measurements compared to HIIT_LIN_ as we hypothesized.

Speed is an important determinant of successful performance in elite female soccer, especially during crucial match actions and off the ball running [23]. We observed similar improvements in acceleration (10 and 20 m) and maximal speed (30 m) in both groups, with increases in the HIIT_LIN_ (5.3–9.7%) and HIIT_COD_ (5.4–8.7%) groups. The differences in training adaptations were unclear between both groups. The magnitude of response was higher for all linear tests compared to the previous study [10] investigating the effects of mixed-methods HIIT intervention in young female soccer players. We observed large and medium average improvements in terms of speed, which is in accordance with Taylor et al. [19], who also observed effects after two weeks of HIIT and reported a large effect (3.6–9.6%) on the acceleration phase of sprinting in male soccer players. Furthermore, the mentioned study had a training programme similar to ours and also had the same linear and COD group. Based on the results, it is possible that the players in our study were less habituated to the training stimulus imposed by the HIIT approach than those in the above-mentioned earlier trials. In addition, based on participants’ age (20 years in our study vs. 13 years), the heterogeneous results suggest that growth and maturation could be significant mediators when measuring physical fitness [24]. Our training programme was designed so that the training load was gradually increased, which was different from the above-mentioned studies. Moreover, the programme was not designed for a specific position. Having this in mind, Mäkiniemi et al. [25] reported position-specific differences in match demands, especially for the distance and number of acceleration and decelerations.

It is well documented that HIIT can improve COD in soccer [15, 19]. Our findings have shown that both training interventions (HIIT_LIN_ and HIIT_COD_) resulted in similar positive changes in players’ ability to change direction. In addition, the magnitude of response in all three measured agility tests for HIIT_LIN_ were from 5.6% to 6.7%, while for HIIT_COD_ they were from 3.9% to 8.2%. The biggest changes were observed in the Zig-zag test in both groups (HIIT_LIN_ 6.7%, HIIT_COD_ 8.2%). The current results are slightly better than those obtained by Nayıroglu et al. [15], who reported a significant improvement (3.5%) in the 505 test. In addition, Wright et al. [10] observed moderate improvements in the T-test in two groups (before peak height velocity (PHV) 2.4%; after PHV 2.4%). Given the design of the experimental programme, we expected that the HIIT_COD_ group would outperform HIIT_LIN_, particularly in the change of direction tests. It should be taken into account that COD volume and their different turn angles during a training session were determinants for adaptations to occur [26]. The rapid improvement seen in the participants’ performance could be explained by the different physiological changes, such as rapid adaptation, muscle fibre recruitment, and frequency and motor unit synchronization [27]. Hence, having in mind that our participants showed far more COD changes compared to the other mentioned studies, our results are understandable.

Improving soccer-specific strength, which is defined as a player’s ability to employ muscle strength, power efficiency and consistent soccer-specific activities throughout the game, should be one of the most essential goals of training programmes [28]. After 6 weeks of HIIT, our programmes had improved the vertical jump in all three variables in both experimental groups. In addition, the magnitude of response in all three measured tests for HIIT_LIN_ ranged from 8.0% to 9.9%, while for HIIT_COD_ it ranged from 10.0% to 13.7%. In this regard, our findings are consistent with those of other soccer-related studies, which reported a significant improvement in CMJ (10.3%) and CMJA tests (10.2%) after eight weeks of HIIT training [15]. Following that, a study with a similar programme and experimental groups to ours reported small improvements in CMJ in the linear (1.5%) and COD (1.8%) groups in men’s soccer [19]. Although we also recorded notable changes in the SJ test in the HIIT_COD_ group (13.7%), our study was also the first to report the effect of HIIT training in female soccer players. The higher number of changes of direction in our training programme could be the primary reason for the disparity of results. Likewise, participants’ sample characteristics (middle-adolescent vs. young adults, professional vs. amateur) should also be taken into consideration. Additional upper-body muscles and eccentric muscular contractions [29] should not be excluded as a rationale for CMJA, as well as neuromuscular and metabolic factors [27], for SJ. Buchheit et al. [30] stated that as sprinting ability demands increase, the explosive strength also increases, which may be another possible explanation of our results. Increased muscular ability to create greater tension adds more contractile components and utilizes elastic energy, and may contribute to alterations in muscle function during jumping performances [31]. Likewise, based on the significant correlation between 10 m sprint and agility tests (180° and 100° angles) [32], we can assume that this is another possible explanation for our results, since our agility tests consisted of 10 m running distance, with 180° angles (Pro-agility test, 9-6-3-6-9 test) and 100° angles (Zig-zag). We can also assume that our results would have been even greater if we had conducted unilateral jump tests, but, unfortunately, we did not. In regard to the mentioned facts, there could be a slight connection, because the COD participants probably performed their agility turns on their preferred leg. Hence, we can speculate that this is a possible cause of our observed improvements in leg power.

Repeated sprint ability consists of a series of brief sprints separated by a short rest period [27], which is an important overall factor in soccer [19]. We observed improvements in both RSA(avg) (HIIT_LIN_-5.1%; HIIT_COD_ -4.7%) and RSA(best) (HIIT_LIN_-0.6%; HIIT_COD_-0.8%). There is a notable study disparity between the results from the study by Wright et al. [10] and the results from the current study. The mentioned study reported small decrements in RSA performance, with moderate individual differences. The RSA outcome was most likely similar to the related ability to develop maximum speed [33]. According to them, the magnitude of accelerations and the amount of time spent accelerating in each running bout are both important; therefore it appears reasonable to believe that these athletes performed more forceful accelerations and spent more time accelerating per running bout. Kaplan [34] did not find any significant differences in RSA, best time, average time and FI, while our study showed the opposite. One of the reasons is that they were evaluating these parameters based on the players’ position, while we did not include that factor. Likewise, their sample consisted of amateur players, while ours consisted of elite players. Several studies [35, 36] have immediate practical implications in that, for both the development of fatigue-reduction methods and a better understanding of the physiological responses, repeated high-intensity exercises are to be followed. In addition, the programme was conducted in the preparation period, designed so that the training load was gradually increased; hence, the improved overall results were also logical. High-quality and accurate results enable the establishment of precise targets, tasks, and preparation cycles, as well as the implementation of appropriate means, loads, and methods for training [37].

Due to the fact that our HIIT programmes were used in preseason, the greatest improvements were expected for aerobic capacity. This was confirmed with large improvements in both groups in V̇O_2max_ (HIIT_LIN_-3.9%; HIIT_COD_-7.4%) and VIFT (HIIT_LIN_-4.0%; HIIT_COD_-7.8%) without significant differences between them. The results from the current study can be related to the results from the previous studies [8, 9, 15, 38]. According to Savolainen et al. [39], the total distance covered in a match could be increased by 1450 m as a result of the players’ V̇O_2max_ improvement in an elite level by 10 ml/kg/min. In accordance with that, Rowan et al. [9] reported a significant increase in V̇O_2max_ (3.4–4.7%), while Arazi et al. [8] noted large improvements in the same variable after 6 weeks of an experimental programme (12.8%). Moreover, when VIFT was taken into account, we identified improvements in both groups. However, further discussion is limited due to the restricted number of studies in this area of investigation. Although the previous research has pointed out that HIIT alone can lead to the improvement of VIFT [40], we found that greater effects of improvement were observed in the COD group. This finding is congruent with the work of Nayıroglu et al. [15], who noted significant improvements (10.7%). In addition, Dolci et al. [38] reported significant differences after two weeks of HIIT in both groups (4.0% and 5.0%, respectively). A possible explanation for our results could be given by Taylor et al. [19], who suggested that incorporating COD activities into training leads to greater metabolic and neuromuscular stress, adaptation and better performance at the same time. In addition, Padulo et al. [41] stated that including specific COD movements/turns into a training programme may have better positive transfer to real game situations than linear running. Also, taking into account that VIFT is associated with changes in several variables, of which the most important are V̇O_2max_ and COD activities, it can be concluded that the improved VIFT values are also the result of the experimental treatment. More precisely, the same muscle groups are involved in a large number of sudden decelerations and accelerations during 30–15 IFT [30]. Furthermore, repeating the same patterns of these movements during HIIT training leads to positive changes and a positive transfer to the performance. Knowing the demands of elite women’s soccer matches can be of great value not only to head coaches, but also to physical coaches, in order to plan tailor-made training [42].

We acknowledge that our study had some limitations. Firstly, our study is not generalizable beyond the study sample due to the wide range of age of the participants (16–26 years). Secondly, we failed to examine the effects of two different types of HIIT with the presence of the control group due to the lack of an adequate sample of the participants. Nevertheless, this is the first study aimed at comparing two different types of HIIT training (LIN-COD), especially in the highest rank of the competition, and produced positive results. In future studies will be necessary to further investigate the role of different types of HIIT training in female soccer performance.

## CONCLUSIONS

Based on the obtained results, we can conclude that different types of HIIT training, i.e., both LIN and COD, have a positive effect on physical performance in elite female soccer player. According to our research, the importance and effects of training that includes a change in direction of movement, which represents more specific movements similar to soccer, can be achieved. This study should be understood as a good practical framework, in order to level up the physical performance variables in the preparation period, as well as to achieve the maximum results. To conclude, these findings would be useful to coaches and sports scientists, who should include a specially created HIIT training programme for each player in particular and in the micro cycle as a powerful method of improving their performance.
